# An Environmentally Relevant Mixture of Perfluorooctanesulfonic Acid and Perfluorohexanesulfonic Acid Does Not Conform to Additivity in Northern Leopard Frogs Exposed Through Metamorphosis

**DOI:** 10.1002/etc.5486

**Published:** 2022-11-02

**Authors:** Tyler D. Hoskins, Elizabeth B. Allmon, R. Wesley Flynn, Linda S. Lee, Youn Choi, Jason T. Hoverman, Maria S. Sepúlveda

**Affiliations:** ^1^ Department of Forestry and Natural Resources Purdue University West Lafayette Indiana USA; ^2^ Department of Agronomy Purdue University West Lafayette Indiana USA; ^3^ Sustainability Research Center, Life Sciences Faculty Universidad Andres Bello Santiago Chile

**Keywords:** Perfluoroalkyl substance, PFAS, bioaccumulation, amphibians, mixtures

## Abstract

Per‐ and polyfluoroalkyl substances (PFAS) are chemicals associated with adverse health effects. At aqueous film–forming foam sites, they occur as mixtures, with perfluorooctanesulfonic acid (PFOS) and perfluorohexanesulfonic acid (PFHxS) commonly co‐occurring in the highest concentrations. Although PFOS and PFHxS toxicities have been studied, few studies have tested their potential interaction. Using *Rana pipiens*, the present study compared toxicities of a 1:1 PFOS:PFHxS mixture to PFOS and PFHxS individually with the prediction that responses would be additive. Gosner stage 25 (GS 25) tadpoles were exposed through metamorphosis (GS 46) to 0.5 and 1 ppb PFOS or PFHxS alone or to a mixture of 0.5 ppb PFOS and 0.5 ppb PFHxS. Tadpoles were weighed and measured (snout‐vent length [SVL]) at day 31, metamorphic climax (GS 42), and GS 46. These values were used to calculate the scaled mass index (SMI), a measure of body condition. Body burdens were quantified on day 31 and at GS 46. The PFOS and PFHxS body burdens were elevated relative to controls at GS 46. No effects were observed on survival, SVL, or mass. Single PFAS effects included a 17% reduction in SMI at day 31 (0.5 ppb PFHxS) and a 1.1‐day longer metamorphic period (1 ppb PFHxS) relative to controls. Mixture results deviated from additivity—SMIs were higher than expected on day 31 and lower than expected at GS 42. In addition, time to GS 42 in the PFAS mixture exceeded expected additivity by 12 days. Results from a chronic exposure to a 1:1 PFOS:PFHxS mixture resulted in changes in body condition and length of metamorphosis that deviated from additivity. More PFAS mixture toxicity studies conducted at relevant ratios and concentrations are needed. *Environ Toxicol Chem* 2022;41:3007–3016. © 2022 The Authors. *Environmental Toxicology and Chemistry* published by Wiley Periodicals LLC on behalf of SETAC.

## INTRODUCTION

Poly and perfluoroalkyl substances (PFAS) are synthetic persistent chemicals commonly found in the environment as mixtures. One source of PFAS to the aquatic environment are facilities that use(d) aqueous film–forming foams (AFFFs) to contain fuel‐based fires such as airports and military bases (Anderson et al., 2016; East et al., 2020). In a recent study, perfluorooctane sulfonate (PFOS) and perfluorohexane sulfonate (PFHxS) were found at the highest concentrations in surface water from 256 AFFF‐impacted sites sampled across 85 installations in the United States and, together with the carboxylic acids perfluorohexanoic acid (PFHxA) and perfluorooctanoic acid (PFOA), accounted for >80% of the sum of all PFAS in the samples tested (East et al., 2020). The concentrations of PFOS and PFHxS were similar across sites, with overall means of 0.25 and 0.23 µg/L, respectively. Median concentrations across the installations, however, deviated from a 1:1 relationship and were 0.62 PFHxS:1 PFOS. Therefore, at AFFF‐impacted sites, biota are exposed to a mixture of PFAS that is primarily composed of PFOS and PFHxS.

The vast majority of PFAS toxicity data have been derived from single‐compound studies (mostly PFOA and PFOS), with a very small number of studies evaluating the toxicity of PFAS mixtures. For recent reviews on PFAS mixture toxicity, readers should refer to Ankley et al. (2021), and Goodrum et al. (2021). Exposure to PFAS in nature occurs via mixtures that vary in their halogenated carbon chain length and their functional head groups. Both factors are known to directly impact half‐lives and potency. For example, longer‐chain (C8) and sulfonated PFAS are more bioaccumulative and toxic compared with shorter‐chain (C6) and carboxylated forms (Agency for Toxic Substances and Disease Registry, 2018). However, data from recent studies support the fact that PFAS mixture toxicity is only partially driven by the physicochemical properties of the PFAS under study because toxicity outcomes are widely variable and difficult to predict, particularly at the whole‐animal level. Indeed, the few in vivo PFAS mixture studies available report additive (Bursian et al., 2020; Dennis et al., 2020; Flynn et al., 2019), antagonistic (Dennis et al., 2021; Menger et al., 2020), and synergistic (Yang et al., 2019) responses, depending on the animal model, length of exposure, dose, developmental stage, mixture components, and endpoints considered. Furthermore, some studies report more than one type of interaction depending on the ratio of the binary mixtures tested (Ding et al., 2013). There is little understanding of the mechanisms behind these apparently contradictory responses.

Binary mixture in vivo toxicity studies with PFOS and PFHxS are restricted to two studies. Northern bobwhite quail (*Colinus virginianus*) were exposed to PFOS and PFHxS, and synergism (increased hatching success compared to controls) was found in birds dosed with 0.958 ppb total PFAS at a PFOS:PFHxS ratio of 1.8:1 or to 22.9 ppb total PFAS at a PFOS:PFHxS ratio of 1.2:1 (Dennis et al., 2020). However, for other endpoints, such as body weight in adult females, there was a significant decline in the high PFOS:PFHxS treatment. The authors conclude that the interaction between PFOS and PFHxS leads to different responses compared with single PFAS, which would suggest different modes of toxicity. In the second study, McCarthy et al. (2021) tested a binary mixture of 2.5 µg/L PFOS + 1000 µg/L PFHxS for 20 days using the water midge *Chironomus dilutus* and reported decreased survival in the mixture compared to controls. The authors also suggest a potential additive or synergistic interaction between PFOS and PFHxS.

Previous work testing PFAS mixtures in amphibians points toward additivity. For example, the inhibition concentration was modeled from in vitro data collected using a *Xenopus tropicalis* fibroblast cell line exposed to different binary mixtures of PFAS (PFOS + PFOA, PFOA + PFHxA, PFOA + PFHxS, and PFOS + PFHxS; Hoover et al., 2017). All binary mixture combinations showed concentration addition, suggesting a similar mechanism of action for all considered PFAS (Kar et al., 2017). Importantly, additional studies with developing American bullfrog, *Rana catesbeiana*, larvae also showed that exposure to a binary PFAS mixture (PFOS + PFOA) results in developmental delays that do not deviate from additivity (Flynn et al., 2019). Further, results from an exposure of northern leopard frogs, *Rana pipiens*, throughout metamorphosis to a complex mixture resembling an AFFF site also support additivity (T. D. Hoskins et al., unpublished data).

Recognizing that the binary mixture PFOS + PFHxS is highly representative of surface waters from AFFF‐impacted sites, representing approximately 80% of a “typical” AFFF surface water sample, we chronically exposed northern leopard frogs to a PFOS + PFHxS 1:1 mixture (0.5 ppb each) and evaluated effects on growth and development. We hypothesized that chronic exposure to PFOS and PFHxS would result in concentration‐dependent declines in body condition and delayed development and that these responses would not deviate from additivity when animals were co‐exposed to both. We also hypothesized that changes in growth and development would be more pronounced in the longer‐chain PFOS (C8) compared with the shorter‐chain PFHxS (C6) treatments. To our knowledge, our study is the first to test an environmentally relevant PFOS + PFHxS mixture in amphibians exposed throughout larval development, including metamorphosis.

## MATERIALS AND METHODS

### Test chemicals and stock preparation

Stock solutions were prepared using technical‐grade PFAS (Sigma‐Aldrich; lot numbers BCBR8860V, ≥98% purity, and BCBW1770, ≥98% purity, for PFOS and PFHxS, respectively). Stocks were prepared in polypropylene bottles by adding either 500 mg PFOS or 1000 mg PFHxS to 1 L Ultrapure (Milli‐Q) water. Solutions were stirred on a magnetic plate at room temperature for 24 h prior to use.

### Test organisms

The present study was conducted under and in accordance with an approved Purdue Institutional Animal Care and Use Committee protocol (no. 1601001355). In March 2019, northern leopard frog egg masses (*n* = 4) were collected from an ephemeral pond at the Purdue Wildlife Area, Tippecanoe County, Indiana, USA. Egg masses were transferred to individual 200‐L outdoor tanks containing 150 L of aged well water and covered with 70% shade cloth until larvae reached Gosner Stage (GS) 25 (Gosner, 1965). During this time, larvae were fed Purina® Rabbit Chow pellets daily, and water changes were performed every 48 h. A month later, once larvae reached GS 25, they were transported to the laboratory, sorted in trays, and assigned to experimental units.

### PFAS exposure conditions

Tadpoles were acclimated to laboratory conditions for 3 days prior to the start of the experiment, which was conducted between May 10 and September 2 (120 d) at the Purdue Wildlife Area. Treatments consisted of a control, 0.5 ppb PFOS, 0.5 ppb PFHxS, 1 ppb PFOS, 1 ppb PFHxS, and a mixture containing 0.5 ppb PFOS and 0.5 ppb PFHxS, with four replicates each for a total of 24 experimental units. Experimental units consisted of 15‐L polypropylene plastic aquaria, filled with 7.5 L of aged filtered and ultraviolet‐treated well water. Each experimental unit was stocked with 20 haphazardly selected tadpoles. At the initiation of the experiment (day 0) all larvae were at GS 25 and had a mean ± SD snout–vent length (SVL) and body mass of 5.03 ± 0.41 mm and 23.4 ± 5.8 mg, respectively (*n* = 10).

The experiment was conducted using a static renewal system constructed inside a temperature‐controlled room with a complete set of replicates set up in each of four shelves (complete randomized block design). No air was provided to the tanks. Temperature was set at 21 °C on a 12: 12‐h light: dark cycle. Every 48 h, all tadpoles from each experimental unit were gently captured with a mesh net and temporarily transferred into 2‐L polypropylene plastic aquaria, with water kept at the same temperature as treatment aquaria. After proper disposal of exposure media, aquaria were rinsed, filled with 7.5 L of aged well water, and spiked with the target PFAS. On experimental days 86–88, un‐ionized ammonia (NH_3_) concentrations from four aquaria measured between 0.18 and 0.35 mg/L. Thereafter, we reverted to daily water changes.

Feed controls (*n* = 4 tanks) were used to adjust feeding rates throughout our study. These larvae were weighed once weekly, and feeding rates for all experimental animals were calculated as 10% of their average body mass. Tadpoles were fed every other day, immediately following water changes.

### Environmental parameters

Water quality parameters (temperature, dissolved oxygen, pH, conductivity, and ammonia) were measured at least weekly throughout our study (Table 1). Leading up to day 35 of the study, dissolved oxygen levels within tanks were consistently low (<4 mg/L); therefore, on day 35, the population densities of all tanks were reduced to 12 tadpoles per tank to reduce demand for dissolved oxygen within tanks and maintain appropriate water quality (Table 1).

**Table 1:** Water quality parameters (mean ± SEM, minimum, maximum) measured before and after reducing population densities in each tank on day 35 of exposure

Water quality measurements							
		Prior to population reduction			Following population reduction		
	Unit	Mean ± SEM	Min. value	Max. value	Mean ± SEM	Min. value	Max. value
Temperature	°C	20.3 ± 0.1	19.4	20.9	20.3 ± 0.1	19.4	21.0
Dissolved oxygen	mg/L	4.3 ± 0.3	2.4	8.0	5.9 ± 0.3	1.8	8.8
pH		7.87 ± 0.02	7.72	8.10	7.87 ± 0.03	7.40	8.25
Total ammonia nitrogen	ppm	0.72 ± 0.12	0.25	2.00	0.47 ± 0.06	0.00	1.00
NH_3_	ppm	0.03 ± 0.01	0.01	0.08	0.03 ± 0.01	0.01	0.09

### Responses measured

Survival was monitored daily. Animals were sampled at three time points: day 31 (when ≥50% of feed controls reached GS 29), metamorphic climax (GS 42), and tail resorption (GS 46). On day 31, tadpoles (*n* = 5) were haphazardly selected from each experimental unit and euthanized in buffered tricaine methanesulfonate at 3 g/L, gently blotted on paper towels to remove excess water, weighed, measured (SVL), and staged following published protocols (Gosner, 1965). Scaled mass index (SMI) was calculated using mass and SVL such that

$${\hat{M}}_{i}=M_{i}{\left[\frac{L_{0}}{L_{i}}\right]}^{b_{SMA}}$$

where *M*_*i*_ and *L*_*i*_ are the mass and length measurement of individual *I*, respectively; *L*_0_ is the mean of the control study population; and *b*_*SMA*_ is the scaling exponent estimated by the standardized major axis (SMA) regression calculated as a slope of reduced major axis regression of log‐transformed body length on log‐transformed body mass (Peig & Green, 2009).

Remaining tadpoles were reared in their respective experimental units to metamorphic climax (GS 42). On reaching GS 42, metamorphs were processed for weight and SVL, SMI was calculated as described above, and metamorphs were subsequently reared individually in polypropylene deli cups (11.4 cm diameter at base; Fabri‐Kal®) with approximately 1 cm of water from their respective experimental units). Containers were placed at an angle and covered with a perforated lid, providing both a dry area for emergence and a wet area for rehydration. Metamorphs were monitored daily until they reached tail resorption (GS 46). No water changes occurred during this time. On reaching GS 46, metamorphs were euthanized and processed for phenotypic endpoints as described above. The aquatic PFAS exposure period was terminated on day 120, at which point any tadpoles still alive and yet to reach GS 42 were collected for phenotypic measurements and euthanized as described above.

### Chemical analyses

Concentrations of PFAS in test water and whole tadpoles were quantified using previously validated liquid chromatography tandem mass spectrometry (LC‐MS/MS) analytical methods (Flynn et al., 2019; Flynn, Iacchetta, et al., 2021; Hoover et al., 2017). Data were acquired using an ultra‐high performance liquid chromatograph (HPLC) MS system coupled with a 8040 Shimadzu MS. Acquisition mode was done under negative electrospray ionization and multiple reaction monitoring mode.

One water sample (10 mL) was analyzed for both PFOS and PFHxS from each replicate within treatments (*n* = 4/treatment, total *n* = 24) at experimental teardown and frozen at −20 °C until processed by the analytical chemistry laboratory at Purdue University. Chemical analyses of tadpole body burdens were conducted on tadpoles collected on day 31 and at GS 46 (*n* = 2 per experimental unit, *n* = 8 per treatment). Individuals collected for body burden analysis were stored in 15‐ml polypropylene tubes and frozen at −20 °C until lyophilized. Next, 3:1 v/v tetrahydrofuran/nanopore water (total 800 µl) was added to 0.05–0.2 g freeze‐dried samples in 1.5‐mL polypropylene microcentrifuge tubes. After adding 10 ng of each isotopically mass labeled PFAS, samples were extracted by vortexing (1500 rpm for 10 min) and sonicating (30 min), followed by centrifugation. The supernatant was transferred for evaporation under gentle N2 flow and reconstituted in 1:1 v/v MeOH/H_2_O solution. After vortexing, sonicating, and centrifuging the reconstituted sample, the supernatant was retransferred into an HPLC vial for analysis by LC‐MS/MS. Instrument running conditions included Kinetex® 5‐µm EVO C18 (Phenomenex; 100 Å, LC column 100 × 2.1 mm) with a guard filter (Phenomenex; KrudKatcher ULTRA HPLC in‐line filter, 2.0 µm depth filter × 0.004 in inner diameter) with column temperature maintained at 40 °C. A combined flow rate of 0.4 mL/min was used for chemical separation with mobile phases A (20 mM ammonium acetate in water) and B (methanol). Starting at 5% B and maintaining for 0.2 min, the gradient increased to 30% B by 2 min, then to 100% by 13.6 min, maintained for 3.4 min, and back down to 5% in 0.1 min for equilibrium. The total running time was 21.5 min, and the calibration range was between 0.01 and 50 µg/L in 1:1 v/v MeOH/H_2_O. More details on the analytical methods used, including limits of detection and limits of quantification are available in Supporting Information, Table S1.

Bioconcentration factor (BCF) values were calculated for GS‐46 tadpoles (*n* = 1–2 per experimental unit, *n* = 6–8 per treatment) for PFOS and PFHxS. The BCFs were calculated by dividing the body burden of the target PFAS of GS‐46 frogs by the concentration of the target PFAS in the water at experimental teardown.

### Statistical analyses

All statistical analyses were performed in Sigma Plot, Ver 13.0. Concentrations of PFAS in water were compared across treatments using one‐way analysis of variance (ANOVA). Survival, body burdens, and phenotypic endpoints (GS, SVL, mass, SMI) measured across all time points were analyzed using one‐way ANOVA with a fiducial level of significance of *p* ≤ 0.05. Post hoc comparisons versus controls were analyzed using Dunnett's tests, and planned comparisons defining differences within chemical treatment (i.e., 0.5 ppb PFOS vs. 1 ppb PFOS) or PFAS concentration (i.e., 0.5 ppb PFOS vs. 0.5 ppb PFHxS) were conducted using Holm‐Sidak post hoc tests. Expected values based on additivity were calculated for each PFAS (PFOS 0.5 ppb and PFHxS 0.5 ppb) by determining their relative difference from the controls. Those differences were then summed to derive the expected response difference assuming additivity for the mixture treatment (Billet et al., 2020). Paired *t* tests were used to compare observed and expected values of PFOS and PFHxS additivity within the mixture treatment.

## RESULTS

### Environmental conditions

Room temperature during the experiment was 21.8 ± 0.6 °C (mean ± SE), and water temperature remained stable at 20.3 ± 0.1 °C. Prior to reducing population densities in tanks at day 35, dissolved oxygen levels averaged 4.3 ± 0.3 mg/L. Following population density reduction to *n* = 12 tadpoles per tank, dissolved oxygen levels increased to 5.9 ± 0.3 mg/L. A summary of water quality parameters quantified is presented in Table 1. Except for a spike in NH_3_ at approximately day 90, which was immediately remediated with a water change, all parameters stayed within acceptable levels.

### PFAS in water and tadpoles

All PFAS exposure treatments were dosed near nominal concentrations. The PFAS water chemistry data (mean ± SE, minimum, maximum, and lower limit of quantification values) for PFOS and PFHxS across all treatments are listed in Table 2.

**Table 2:** Analytical water chemistry values (mean ± SEM, minimum, maximum) for perfluorooctanesulfonic acid and perfluorohexanesulfonic acid across all treatments

Analytical water chemistry					
	Units	Chemical	Mean ± SEM	Min. value	Max. value
Control	µg/L	PFOS	ND	–	–
		PFHxS	ND	–	–
PFOS 0.5 ppb	µg/L	PFOS	0.557 ± 0.094	0.402	0.829
		PFHxS	ND	–	–
PFOS 1 ppb	µg/L	PFOS	0.934 ± 0.026	0.866	0.987
		PFHxS	ND	–	–
PFHxS 0.5 ppb	µg/L	PFOS	ND	–	–
		PFHxS	0.445 ± 0.023	0.407	0.504
PFHxS 1 ppb	µg/L	PFOS	ND	–	–
		PFHxS	1.183 ± 0.009	1.166	1.207
Mixture	µg/L	PFOS	0.441 ± 0.061	0.293	0.584
		PFHxS	0.550 ± 0.010	0.528	0.568

PFOS = perfluorooctanesulfonic acid; PFHxS = perfluorohexanesulfonic acid; ND = not detected.

**Figure 1:**
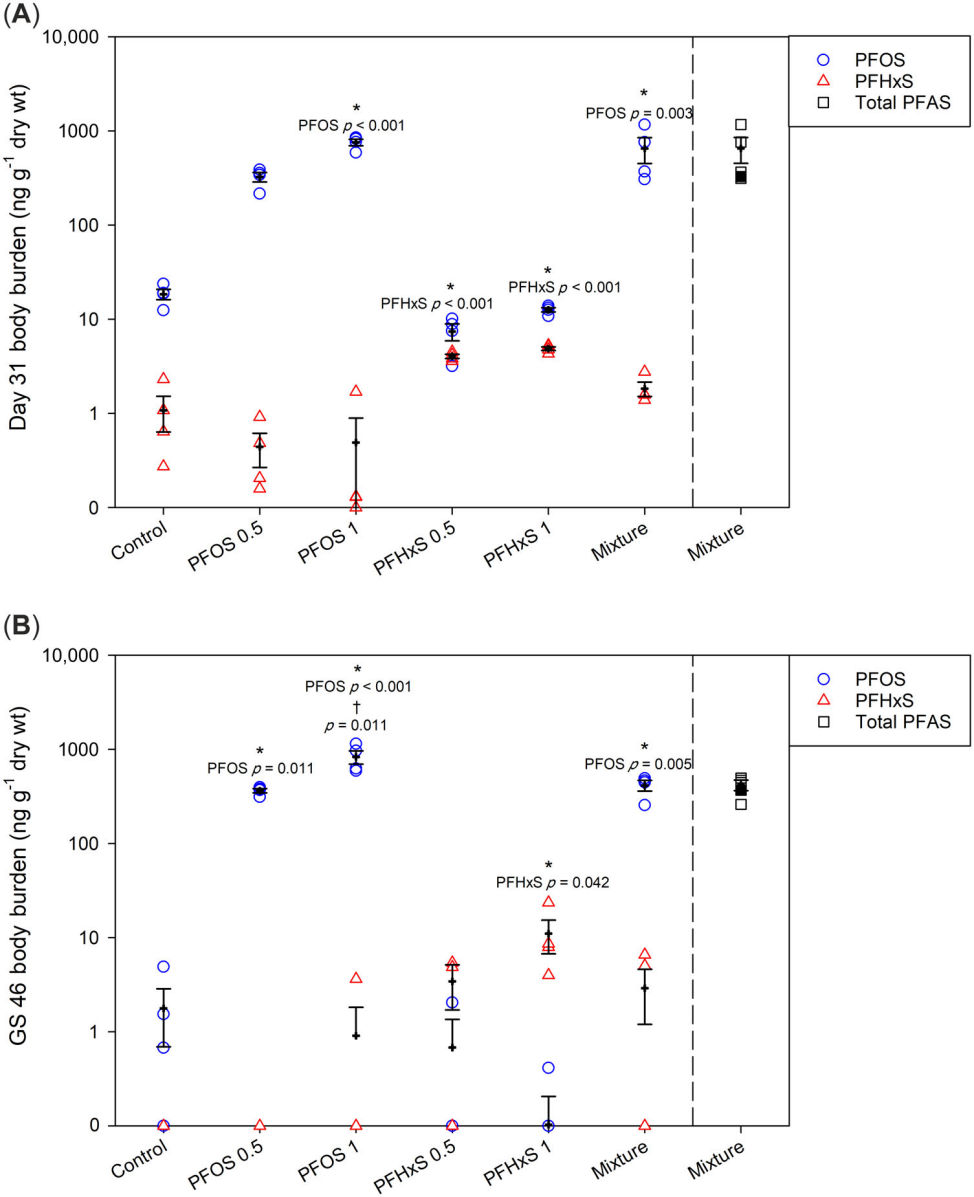
Perfluorooctanesulfonic acid (PFOS; blue circles) and perfluorohexanesulfonic acid (PFHxS; red triangles) body burdens (mean ± SEM) in northern leopard frog tadpoles (**A**) at day 31 and (**B**) at end of metamorphosis following exposure to PFOS, PFHxS, or a mixture of PFOS and PFHxS. Total additive perfluoroalkyl substance (PFAS) body burdens (mean ± SEM) are shown in black with shaded points representing expected total PFAS body burdens based on additive effects of PFOS and PFHxS at 0.5 ppb at each time point. *Significant differences from controls within each chemical (*p* ≤ 0.05). ^†^Significant differences between doses of the same chemical (*p* ≤ 0.05). GS = Gosner stage.

Concentrations of PFAS in whole tadpoles at day 31 are shown in Figure 1A and summarized in Supporting Information, Table S2A. Control tadpoles were found to contain both PFOS (mean 18.5 ppb) and PFHxS (1.1 ppb), and potential exposure sources are discussed below. For the 0.5‐ and 1‐ppb treatments, PFOS bioaccumulated to a mean of 325 and 755 ppb, respectively. In the mixture group, PFOS body burdens reached a mean of 651 ppb, almost twice as much compared to the 0.5‐ppb PFOS single treatment. For PFHxS, body burdens reached 4 and 4.9 ppb for the low and high treatments, and both concentrations were higher compared to controls. Interestingly, tadpoles coexposed to 0.5 ppb each of PFHxS and PFOS had PFHxS body burdens that were only 40% of those exposed to only 0.5 ppb PFHxS. Moreover, whole‐larvae concentrations of PFHxS in the mixture (1.8 ppb) were not significantly different from controls. Full statistical summaries of day 31 body burden data can be found in Supporting Information, Table S3A.

The PFAS body burdens at completion of metamorphosis (GS 46) are shown in Figure 1B and summarized in Supporting Information, Table S2C. By the end of the experiment, PFAS contamination in controls was significantly less: PFOS was detected in very small amounts (mean 1.8 ppb), and PFHxS was not detected. The PFOS body burdens in treatment animals were significantly higher than in controls and again increased in a dose‐dependent manner (from 363 to 829 ppb for the 0.5‐ and 1‐ppb treatments, respectively). In addition, there was no difference in PFOS body burdens in animals exposed to the PFAS mixture (413 ppb) and those exposed to 0.5 ppb PFOS. For PFHxS, exposure to 0.5 and 1 ppb resulted in mean body burdens of 3.42 and 11.03 ppb (one‐way ANOVA and Dunnett's, *p* = 0.063 and *p* = 0.042, respectively). Exposure to the PFAS mixture resulted in nonsignificant differences in PFHxS body burdens relative to controls (one‐way ANOVA and Dunnett's, *p* = 0.140). Full statistical summaries of body burdens at completion of metamorphosis can be found in Supporting Information, Table S3C.

The PFAS BCFs were calculated for animals sampled at GS 46 and are presented and discussed as log10‐transformed values from the concentrations shown in Supporting Information, Table S2C. For PFOS, BCFs were 2.88 for the 0.5‐ppb group and 3.09 for the 1‐ppb group. In the mixture, PFOS BCF fell within this range (3.09). In contrast, PFHxS did not bioconcentrate (BCFs 1.07–1.30).

### Phenotypic effects

Survival across the experimental period from GS 26 to GS 46 ranged between 69% and 79% (Supporting Information, Table S2C) and was not affected by treatment (Supporting Information, Table S3C). In addition, no relationship was observed between average tank survival and any phenotypic endpoint (data not shown). Full summaries of all phenotypic endpoints measured across the experiment (mean ± SEM, minimum, and maximum values) can be found in Supporting Information, Table S2. Full statistical summaries of all comparisons of phenotypic endpoints can be found in Supporting Information, Table S3.

**Figure 2:**
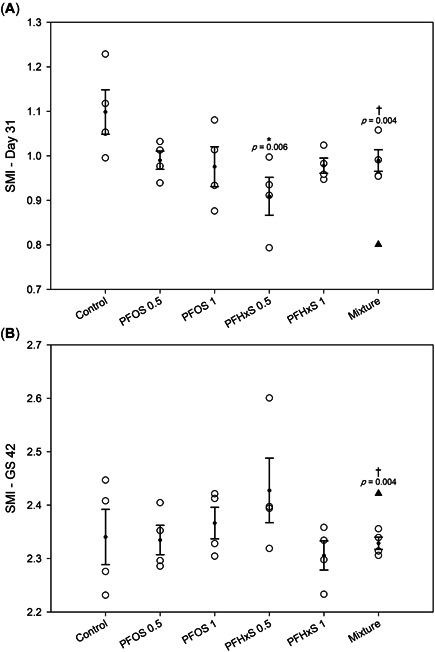
Scaled mass index (SMI; mean ± SEM) of northern leopard frog tadpoles (**A**) at 31 days of exposure and (**B**) at metamorphic climax (Gosner Stage 42). Shaded triangles within the mixture treatments represent the expected SMI based on additive effects of perfluorooctanesulfonic acid and perfluorohexanesulfonic acid at 0.5 ppb at each time point. *Significant differences from controls (*p* ≤ 0.05). ^†^Significant differences from the expected additive effects within the mixture treatment (*p* ≤ 0.05). PFOS = perfluorooctanesulfonic acid; PFHxS = perfluorohexanesulfonic acid; GS = Gosner stage.

On day 31, there were no significant impacts of PFAS treatment on development (measured as GS), SVL, or body mass (Supporting Information, Table S3A). However, SMI was significantly reduced in the PFHxS 0.5‐ppb treatment relative to the control (Figure 2A; Supporting Information, Table S3A). In addition, the observed SMI of the PFAS mixture treatment was significantly higher than the expected SMI based on additivity (Figure 2A; Supporting Information, Table S3A).

Exposure to PFAS had no effect on the SVL, mass, or SMI of tadpoles at metamorphic climax (GS 42) across all treatments relative to the control (Figure 2B; Supporting Information, Table S3B). In addition, PFAS had no effect on survival to GS 42 or the time it took animals to reach this stage (Supporting Information, Table S3C). The first animals to reach GS 46 and complete metamorphosis were detected on day 72 of the exposures; the last animals to complete metamorphosis did so on day 120. Time to metamorphic climax in the PFAS mixture treatment (89.7 days) was significantly longer than the expected time (77.6 days), assuming additive effects (Supporting Information, Table S3B). Contrary to trends seen on day 31, the observed SMI of the PFAS mixture treatment was significantly reduced relative to the expected values at metamorphic climax (Figure 2B).

**Figure 3:**
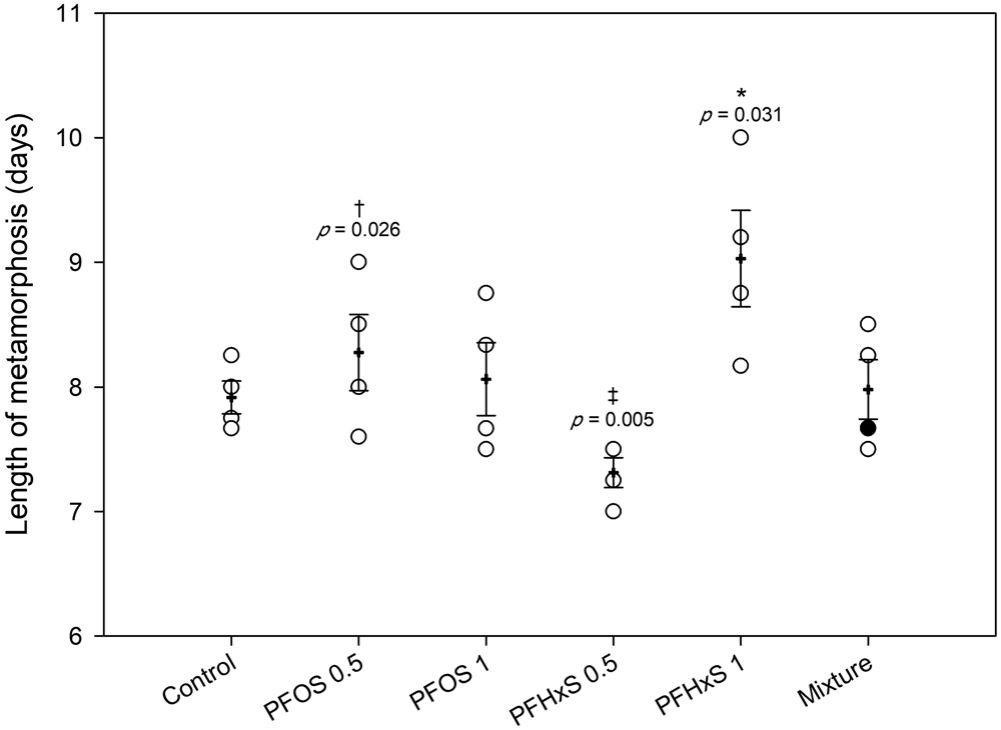
Length of metamorphosis (mean ± SEM) of northern leopard frog tadpoles across all treatments. Shaded point within the mixture treatment represents the expected length of metamorphosis based on additive effects of perfluorooctanesulfonic acid and perfluorohexanesulfonic acid at 0.5 ppb. *Significant differences from controls (*p* ≤ 0.05). ^†^Significant differences between chemical treatments at the same dose (*p* ≤ 0.05). ^‡^Significant differences between doses of the same chemical (*p* ≤ 0.05). PFOS = perfluorooctanesulfonic acid; PFHxS = perfluorohexanesulfonic acid.

Exposure to PFAS impacted the length of metamorphosis (length of time from GS 42 to GS 46; Figure 3). Exposure to 1 ppb PFHxS significantly increased the length of metamorphosis relative to controls by 1.1 days, from a mean of 7.9 to 9.0 days. This response was dose‐dependent, with exposure to 1.0 ppb resulting in a significantly longer metamorphosis compared to 0.5 ppb. In addition, at the same concentration of 0.5 ppb, PFOS had more of an impact on this parameter relative to PFHxS (8.3 vs. 7.3 days, respectively; Figure 3; Supporting Information, Table S3C). Interestingly, there was no difference in the change in SMI across metamorphosis in any treatment. The observed change in SMI also did not differ from the expected additive change in SMI across metamorphosis (Supporting Information, Figure S1).

Although there were some tadpoles (*n* = 16–19 per treatment) that never reached GS 42 during the 120‐day exposure period, there were no differences in the proportion of the population that did not reach metamorphic climax among treatments (Supporting Information, Table S3D). Similarly, there were no differences in the GS of the nonmetamorphosed tadpoles across treatments (Supporting Information, Table S3D).

## DISCUSSION

In the present study, we exposed northern leopard frogs to PFOS or PFHxS singly (0.5 and 1 ppb) or in a mixture (0.5 ppb PFOS + 0.5 ppb PFHxS). Larvae were exposed for up to 120 days, from GS 25 through tail absorption (GS 46). We hypothesized that exposure to PFOS and PFHxS would result in concentration‐dependent declines in body condition and delayed development and that these responses would not deviate from additivity. We also hypothesized that changes in growth and development would be more pronounced in the longer‐chain PFOS (C8) compared with the shorter‐chain PFHxS (C6) PFAS. In contrast, we observed that the effects of the binary mixture did not conform to additivity, that a higher PFAS concentration did not always result in increased toxicity, and that PFOS was not more toxic than PFHxS.

### PFAS body burdens and BCFs

In controls, PFAS were detectable but at very low concentrations compared with PFAS‐exposed animals. For example, on day 31, controls had on average 18–41 times less PFOS compared to tadpoles exposed to 0.5 ppb and 1 ppb PFOS, respectively, with a maximum value of 23.7 ppb detected in one animal. By GS 46, these differences had grown to 200‐ and 460‐fold, respectively, compared to controls. Also, PFHxS was only detected in the controls during the first sampling event. We believe the source of exposure came mostly from the food because an analysis of two Tetramin® flake samples contained 12.48 and 8.43 ppb PFHxS and 1.77 and 2.21 ppb PFOS, respectively. Eighteen days after test initiation, Tetramin flakes were dropped as a food source and rabbit chow was adopted for the remainder of our study because it was shown to contain less PFHxS (3.95 and 2.57 ppb PFHxS for two independent rabbit chow samples) and nondetectable levels of PFOS (Y. Choi et al., unpublished data).

As expected, PFOS bioaccumulated, with log10 BCFs ranging from 2.9 to 3.1. This range in BCFs for PFOS is somewhat higher compared with values reported by Abercrombie et al. (2019) after exposing *R*. *pipiens* tadpoles acutely (4 days) to 10 ppb PFOS (BCF = 2.0). In the same study, American toads, *Anaxyrus americanus*, and eastern tiger salamanders, *Ambystoma tigrinum*, exposed to PFOS under the same conditions had BCFs also lower than what is reported in the present study (2.2–2.5). In contrast, PFHxS BCFs were closer to 1. This highly bioaccumulative nature of PFOS is consistent with previous studies in amphibians (Burkhard, 2021). The lack of bioaccumulation potential for the shorter sulfonate PFHxS is also supported by extensive literature which has consistently shown that C8 perfluoroalkyl sulfonates are more bioaccumulative than C6 congeners (Burkhard, 2021). Our data also support previous findings in that BCFs for PFOS were highly dose‐dependent. In other amphibian exposures, increasing dose led to decreasing BCF, but these concentrations tend to be much higher than those used in the present study (≥10 µg/L; Abercrombie et al., 2019; Flynn, Hoskins, et al., 2021; Hoover et al., 2017). In our study, there was a positive relationship between PFOS dose and BCF. Together, these observations support the idea that, at a certain exposure concentration, PFAS receptors within organisms (e.g., carrier proteins like hemoglobin and transthyretin) become saturated and the relationship between dose and BCF can reverse. This further highlights the need for exposures at environmentally relevant concentrations like those in the present study.

We were interested in comparing body burdens after exposure to a binary mixture in relation to the concentrations elicited by single PFAS because chemical interactions during in vivo mixture studies have previously been reported to potentially impact toxicokinetics (Dennis et al., 2021). For instance, in northern bobwhite quail, *Colinus virginianus*, when PFOS was administered alone (18.7 ppb drinking water) it accumulated less in female livers relative to birds exposed to both PFHxS and PFOS (22.9 ppb 1:2.1 ratio). The authors suggested differential absorption and distribution rates in developing embryos, due to differences in protein binding between the two PFAS. Our results are similar on day 31 when whole tadpoles exposed to the binary mixture contained close to twice the amount of PFOS (621 ppb) compared to the single 0.5‐ppb group (325 ppb). However, by the end of the experiment, this difference had decreased considerably. By the end of our study, body burdens of PFHxS in the mixture (5.8 ppb) were not significantly different from the 0.5‐ppb PFHxS group (3.4 ppb). Overall, these results suggest that, at the concentrations tested, coexposure to PFOS and PFHxS appears to have transient effects on bioaccumulation, which disappear after metamorphosis. Clearly, more studies are needed that examine the impact of co‐exposure of PFAS on toxicokinetics and how patterns change through ontogeny and across life‐history transitions like metamorphosis.

### PFAS effects on tadpole growth and development

Exposure to PFAS did not affect survival, SVL, or mass. However, effects on SMI or body condition and time to metamorphosis were observed. On day 31, there was a 17% decline in SMI in the PFHxS 0.5‐ppb animals compared to controls. This decline was transient because by GS 46 it had disappeared, and no differences in SMI across treatments were observed. In amphibians SMI is a reliable indicator of body condition that correlates with energy stores (MacCracken & Stebbings, 2012). It has also been correlated to lipid reserves in amphibians (Brodeur et al., 2020). Therefore, it represents an ecologically relevant endpoint because energy reserves are crucial for long‐term survival and reproduction. These results agree with a previous study on *R. pipiens*, which also reported declines in SMI after PFOS and PFHxS exposure, with a lowest‐observed‐effect concentration (LOEC) of 10 ppb, the lowest concentration tested in that study (Flynn et al., 2022).

The mechanisms responsible for changes in body condition are unknown. As peroxisome proliferator–activated receptor alpha (PPARα) agonists, PFAS may have affected lipid metabolism and overall lipid reserves. They can also disrupt other nuclear receptors involved in lipid homeostasis, including hepatocyte nuclear factor 4‐alpha (Fragki et al., 2021). This is consistent with PFOS and PFHxS mammalian studies reporting disruption in lipid homeostasis by dysregulation of enzymes involved in fatty acid metabolism (Fragki et al., 2021). It is plausible that PFAS are interacting with PPARα and potentially other isoforms of this nuclear receptor in *R. pipiens*, which would be consistent with the literature. Our laboratory is currently testing this hypothesis in a study looking at the effects of PFOS and PFHxS on the PPAR signaling pathway in amphibians because changes in SMI in amphibians exposed to PFAS have been consistently observed in other studies (Abercrombie et al., 2021; Flynn et al., 2022).

Although there were no differences in time to metamorphosis across treatments, the length of metamorphosis (time from GS 42 to 46) was increased in the 1‐ppb PFHxS treatment by approximately 1 day (from 7.9 to 9.0 days). In addition, there was a dose–response increase in length of metamorphosis within the PFHxS treatment after exposure to 1 ppb PFHxS resulting in longer metamorphosis compared to 0.5 ppb PFHxS (7.3 days). Exposure to 0.5 ppb PFOS resulted in a longer metamorphosis (8.3 days) compared to 0.5 ppb PFHxS (7.3 days). Hoover et al. (2017) also reported delayed development in *R*. *pipiens* exposed to PFOS or PFHxS, with a LOEC of 10 ppb for PFHxS and 100 ppb for PFOS. Ankley et al. (2021) tested much higher PFOS concentrations and reported a LOEC for developmental delays of 3000 ppb. Flynn, Hoskins, et al. (2021) found no effects on time to metamorphosis in *R. pipiens* exposed to up to 1000 ppb of PFOS or PFHxS for 30 days, and no effects on time to metamorphosis were observed in *Xenopus laevis* exposed to PFOS (0.1–100 ppb; Cheng et al., 2011). Overall, effects of PFOS and PFHxS on time to metamorphosis in amphibians appear to be small. In relation to our results, a delay of approximately 1 day in the length of metamorphosis is unlikely to be of great biological or ecological significance, especially when not associated with declines in body size, as reported in the present study.

Our results indicate that the 1:1 binary mixture of PFOS and PFHxS deviated from additivity. For instance, on day 31, we observed an antagonistic response in SMI because the predicted mixture toxicity was significantly greater than what was observed. In contrast, at GS 42, the opposite trend was observed, and SMI was lower than expected in the mixture group. A similar type of synergistic effect was observed in the time to reach metamorphic climax (GS 42) in the PFAS mixture treatment, with an approximately 12‐day delay compared with what was expected based on additivity. Deviation from additivity suggests that PFOS and PFHxS induce toxicity through different mechanisms of action. This was proposed in a recent in vivo study with bobwhite quail exposed to three binary mixtures of PFOS and PFHxS (at ratios of 1.2:1 and 1.8:1, with a sum of both equal to 0.375‐, 0.958‐, and 22.9‐ppb exposure concentrations; Dennis et al., 2020). The authors observed that quail responded differently to the binary mixture compared with the single exposures and concluded that the interactive effects observed were the result of different mechanisms of action. A recent in vitro study using human liver cells (HepG2) reported mostly synergism from binary mixture experiments including PFOS and PFHxS, especially at low to medium effect levels (Ojo et al., 2020). Interestingly, these authors found antagonism only in mixtures containing PFOA. In another in vitro system (transiently transfected COS‐1 PPARα reporter), less than additivity with potential for antagonism was reported after testing PFOS and PFHxS (Carr et al., 2013). The authors speculated that it was because both PFAS were competing for activation of the same receptor (PPARα). In the same study, toxicity data fit a concentration addition model but only at the lower concentrations tested. Overall, the limited PFAS mixture toxicity data available points to a complex scenario in which chemical interactions will change depending on a myriad of factors.

There is also an important temporal aspect that needs to be considered when studying the biological responses of PFAS mixtures. The apparent contradictory responses in SMI over time highlight the complexity of studying potential developmental effects in animals undergoing metamorphosis. We had predicted an enhanced sensitivity of PFAS at metamorphosis; however, this was not the case because phenotypic changes were transient and only observed on day 31. It is possible that earlier developmental stages (GS 27–GS 36) are more sensitive to the effects of PFAS than later developmental stages (GS 37–GS 46) and that animals are able to “recover” approximately 3 months later and after an important life‐history transition.

## CONCLUSIONS

Although several studies have attempted to model PFAS mixture toxicity, the results are highly inconsistent and affected by dose, ratios, test duration, test model, developmental stage, and endpoints considered. In addition, the great majority of these studies have been conducted in vitro. The present study investigated whole‐animal sublethal chronic toxicity of a 1:1 mixture of PFOS and PFHxS, the most frequently detected PFAS mixture at AFFF‐impacted sites. We observed that the effects of this binary mixture deviated from additivity, with both antagonism and synergism observed depending on life stage. This is important from a risk‐assessment perspective because an additivity assumption would underestimate or overestimate risk based on synergistic and antagonistic responses, respectively. Therefore, it is critical that more research on PFAS mixture toxicity is conducted to more accurately inform risk. Finally, the maximum PFOS concentration observed in the present study (1231 ppb or 1.231 mg/kg) was below the recently drafted US Environmental Protection Agency's chronic freshwater criterion of 6.75 mg/kg for fish whole body, which should be protective to most aquatic vertebrates and invertebrates (US Environmental Protection Agency, 2022). Our single PFOS toxicity data set supports this value because no major, direct effects were observed in the present study.

## Supporting Information

The Supporting Information is available on the Wiley Online Library at https://doi.org/10.1002/etc.5486.

## Conflict of Interest

The authors declare no conflict of interest.

## Author Contributions Statement

**Tyler D. Hoskins**: Conceptualization; Methodology; Investigation; Formal analysis; Visualization; Writing—original draft. **Elizabeth B. Allmon**: Formal analysis; Visualization; Writing—original draft. **R. Wesley Flynn**: Conceptualization; Formal analysis; Writing—review & editing. **Linda S. Lee, Youn Choi**: Resources; Writing—review & editing. **Jason T. Hoverman**: Conceptualization; Writing—review & editing. **Maria S. Sepúlveda**: Conceptualization; Funding acquisition; Supervision; Writing—original draft.

###  

This article has earned an Open Data badge for making publicly available the digitally shareable data necessary to reproduce the reported results. The data is available at doi: 10.4231/SSV5‐5E21. Learn more about the Open Practices badges from the Center for Open Science: https://osf.io/tvyxz/wiki.

## Supporting information

This article includes online‐only Supporting Information.

Supporting file.

Supporting file.

## Data Availability

All data associated with the present study have been archived through the Purdue University Research Repository (https://purr.purdue.edu/publications/4119/1; DOI: 10.4231/SSV5‐5E21).

## References

[etc5486-bib-1001] Abercrombie, S. A. , de Perre, C. , Choi, Y. J. , Tornabene, B. J. , Sepúlveda, M. S. , Lee, L. S. , & Hoverman, J. T. (2019). Larval amphibians rapidly bioaccumulate poly‐ and perfluoroalkyl substances. Ecotoxicology and Environmental Safety, 178, 137–145. 31002968 10.1016/j.ecoenv.2019.04.022

[etc5486-bib-0001] Abercrombie, S. A. , de Perre, C. , Iacchetta, M. , Flynn, R. W. , Sepúlveda, M. S. , Lee, L. S. , & Hoverman, J. T. (2021). Sublethal effects of dermal exposure to poly‐ and perfluoroalkyl substances on post metamorphic amphibians. Environmental Toxicology and Chemistry, 40, 717–726. 32164037 10.1002/etc.4711

[etc5486-bib-0002] Agency for Toxic Substances and Disease Registry . (2018). Draft toxicological profile for perfluoroalkyls. US Department of Health and Human Services. 37220203

[etc5486-bib-0003] Anderson, R. H. , Long, G. C. , Porter, R. C. , & Anderson, J. K. (2016). Occurrence of select perfluoroalkyl substances at US Air Force aqueous film‐forming foam release sites other than fire‐training areas: Field‐validation of critical fate and transport properties. Chemosphere, 150, 678–685. 26786021 10.1016/j.chemosphere.2016.01.014

[etc5486-bib-0005] Ankley, G. T. , Cureton, P. , Hoke, R. A. , Houde, M. , Kumar, A. , Kurias, J. , Lanno, R. , McCarthy, C. , Newsted, J. , Salice, C. J. , Sample, B. E. , Sepúlveda, M. S. , Steevens, J. , & Valsecchi, S. , (2021). Assessing the ecological risks of per‐ and polyfluoroalkyl substances: Current state‐of‐the science and a proposed path forward. Environmental Toxicology and Chemistry, 40, 564–605. 32897586 10.1002/etc.4869 PMC7984443

[etc5486-bib-0006] Billet, L. S. , Wuerthner, V. P. , Hua, J. , Relyea, R. A. , & Hoverman, J. T. (2020). Timing and order of exposure to two echinostome species affect patterns of infection in larval amphibians. Parasitology, 147, 1–31. 32660661 10.1017/S0031182020001092 PMC10317746

[etc5486-bib-0007] Brodeur, J. C. , Candioti, J. V. , Damonte, M. J. , Bahl, M. F. , Poliserpi, M. B. , & D'Andrea, M. F. (2020). Frog somatic indices: Importance of considering allometric scaling, relation with body condition and seasonal variation in the frog *Leptodactylus latrans* . Ecological Indicators, 116, Article 106496.

[etc5486-bib-0008] Burkhard, L. P. (2021). Evaluation of published bioconcentration factor (BCF) and bioaccumulation factor (BAF) data for per‐ and polyfluoroalkyl substances across aquatic species. Environmental Toxicology and Chemistry, 40, 1530–1543. 33605484 10.1002/etc.5010

[etc5486-bib-0009] Bursian, S. , Link, J. , McCarty, M. , & Simcik, M. (2020). The subacute toxicity of perfluorooctane sulfonate and/or perfluorooctanoic acid and legacy aqueous film‐forming foams to Japanese quail (*Cortunix japonica*) chicks. Environmental Toxicology and Chemistry, 40, 695–710. 32060944 10.1002/etc.4684

[etc5486-bib-0010] Carr, C. K. , Watkins, A. M. , Wolf, C. J. , Abbott, B. D. , Lau, C. , & Gennings, C. (2013). Testing for departures from additivity in mixtures of perfluoroalkyl acids (PFAAs). Toxicology, 306, 169–175. 23470359 10.1016/j.tox.2013.02.016 PMC3810000

[etc5486-bib-0011] Cheng, Y. , Cui, Y. , Chen, H. M. , & Xie, W. P. (2011). Thyroid disruption effects of environmental level perfluorooctane sulfonates (PFOS) in *Xenopus laevis* . Ecotoxicology, 20, 2069–2078. 21809121 10.1007/s10646-011-0749-3

[etc5486-bib-0012] Dennis, N. M. , Karnjanapiboonwong, A. , Subbiah, S. , Rewerts, J. N. , Field, J. A. , McCarthy, C. , Salice, C. J. , & Anderson, T. A. (2020). Chronic reproductive toxicity of perfluorooctane sulfonic acid and a simple mixture of perfluorooctane sulfonic acid and perfluorohexane sulfonic acid to northern bobwhite quail (*Colinus virginianus*). Environmental Toxicology and Chemistry, 39, 1101–1111. 32113193 10.1002/etc.4703

[etc5486-bib-0013] Dennis, N. M. , Subbiah, S. , Karnjanapiboowong, A. , Dennis, M. L. , McCarthy, C. , Salice, J. C. , & Anderson, T. A. (2021). Species and tissue specific avian chronic toxicity values for perfluorooctane sulfonate (PFOS) and a binary mixture of PFOS and perfluorohexane sulfonate. Environmental Toxicology and Chemistry, 40, 899–909. 33210750 10.1002/etc.4937

[etc5486-bib-0014] Ding, G. , Zhang, J. , Chen, Y. , Wang, L. , Wang, M. , Xiong, D. , & Sun, Y. (2013). Combined effects of PFOS and PFOA on zebrafish (*Danio rerio*) embryos. Archives of Environmental Contamination and Toxicology, 64, 668–675. 23479250 10.1007/s00244-012-9864-2

[etc5486-bib-0015] East, A. , Anderson, R. H. , & Salice, C. J. (2020). Per‐ and polyfluoroalkyl substances (PFAS) in surface water near US Air Force bases: Prioritizing individual chemicals and mixtures for toxicity testing and risk assessment. Environmental Toxicology and Chemistry, 40, 871–882. 10.1002/etc.4893 33026654

[etc5486-bib-0017] Flynn, R. W. , Chislock, M. F. , Gannon, M. E. , Bauer, S. J. , Tornabene, B. J. , Hoverman, J. T. , & Sepúlveda, M. S. (2019). Acute and chronic effects of perfluoroalkyl substance mixtures on larval American bullfrogs (*Rana catesbeiana*). Chemosphere, 236, Article 124350. 31319302 10.1016/j.chemosphere.2019.124350

[etc5486-bib-1006] Flynn, R. W. , Hoover, G. , Iacchetta, M. , Guffey, S. , de Perre, C. , Huerta, B. , Li, W. , Hoverman, J. T. , Lee, L. , & Sepúlveda, M. S. (2022). Comparative toxicity of aquatic per‐ and polyfluoroalkyl substance exposure in three species of amphibians. Environmental Toxicology and Chemistry, 41, 1407–1415. 35199880 10.1002/etc.5319 PMC9314107

[etc5486-bib-0018] Flynn, R. W. , Hoskins, T. D. , Iacchetta, M. , de Perre, C. , Lee, L. S. , Hoverman, J. T. , & Sepulveda, M. S. (2021). Dietary exposure and accumulation of per‐ and polyfluoroalkyl substances alters growth and reduces body condition of post‐metamorphic salamanders. Science of the Total Environment, 765, Article 142730. 33077234 10.1016/j.scitotenv.2020.142730

[etc5486-bib-0019] Flynn, R. W. , Iacchetta, M. , de Perre, C. , Lee, L. S. , Sepúlveda, M. S. , & Hoverman, J. T. (2021). Chronic per‐/polyfluoroalkyl substance exposure under environmentally relevant conditions delays development in northern leopard frog (*Rana pipiens*) larvae. Environmental Toxicology and Chemistry, 40, 711–716. 32072676 10.1002/etc.4690

[etc5486-bib-0020] Fragki, S. , Dirven, H. , Fletcher, T. , Grasl‐Kraupp, B. , Bjerve Gützkow, K. , Hoogenboom, R. , Kersten, S. , Lindeman, B. , Louisse, J. , Peijnenburg, A. , Piersma, A. H. , Princen, H. , Uhl, M. , Westerhout, J. , Zeilmaker, M. J. , & Luijten, M. (2021). Systemic PFOS and PFOA exposure and disturbed lipid homeostasis in humans: What do we know and what not? Critical Reviews in Toxicology, 51, 141–164. 33853480 10.1080/10408444.2021.1888073

[etc5486-bib-0021] Goodrum, P. R. , Anderson, J. K. , Luz, A. L. , & Ansell, G. K. (2021). Application of a framework for grouping and mixtures toxicity assessment of PFAS: A closer examination of dose‐additivity approaches. Toxicological Sciences, 179, 262–278. 32735321 10.1093/toxsci/kfaa123 PMC7846094

[etc5486-bib-0022] Gosner, K. L. (1965). A simplified table for staging anuran embryos and larvae with notes on identification. Herpetologica, 16, 183–190.

[etc5486-bib-0023] Hoover, G. M. , Chislock, M. F. , Tornabene, B. J. , Guffey, S. C. , Choi, Y. J. , De Perre, C. , Hoverman, J. T. , Lee, L. S. , & Sepúlveda, M. S. (2017). Uptake and depuration of four per/polyfluoroalkyl substances (PFASs) in northern leopard frog (*Rana pipiens*) tadpoles. Environmental Science & Technology Letters, 4, 399–403.

[etc5486-bib-0024] Kar, S. , Sepúlveda, M. S. , Roy, K. , & Leszczynski, J. (2017). Endocrine‐disrupting activity of per‐ and polyfluoroalkyl substances: Exploring combined approaches of ligand and structure‐based modeling. Chemosphere, 184, 514–523. 28622647 10.1016/j.chemosphere.2017.06.024

[etc5486-bib-0025] MacCracken, J. G. , & Stebbings, J. L. (2012). Test of a body condition index with amphibians. Journal of Herpetology, 46, 346–350.

[etc5486-bib-0026] McCarthy, C. J. , Roark, S. A. , Wright, D. , O'Neal, K. , Muckey, B. , Stanaway, M. , Rewerts, J. N. , Field, J. A. , Anderson, T. A. , & Salice, C. J. (2021). Toxicological response of *Chironomus dilutus* in single‐chemical and binary mixture exposure experiments with 6 perfluoralkyl substances. Environmental Toxicology and Chemistry, 40, 2319–2333. 33835531 10.1002/etc.5066

[etc5486-bib-0027] Menger, F. , Pohl, J. , Ahrens, L. , Carlsson, G. , & Örn, S. (2020). Behavioural effects and bioconcentration of per‐ and polyfluoroalkyl substances (PFASs) in zebrafish (*Danio rerio*) embryos. Chemosphere, 245, Article 125573. 31877453 10.1016/j.chemosphere.2019.125573

[etc5486-bib-0028] Ojo, A. F. , Peng, C. , & Ng, J. C. (2020). Combined effects and toxicological interactions of perfluoroalkyl and polyfluoroalkyl substances mixtures in human liver cells (HepG2). Environmental Pollution, 263, Article 114182. 32247900 10.1016/j.envpol.2020.114182

[etc5486-bib-0029] Peig, J. , & Green, A. J. (2009). New perspectives for estimating body condition from mass/length data: The scaled mass index as an alternative method. Oikos, 118(12), 1883–1891.

[etc5486-bib-0031] Sigma Plot (Version 13.0) [Computer software]. Systat Software.

[etc5486-bib-0032] US Environmental Protection Agency . (2022). Draft aquatic life ambient water criteria for perfluorooctanoic sulfonate.

[etc5486-bib-0033] Yang, H.‐B. , Zhao, Y.‐Z. , Tang, Y. , Gong, H.‐Q. , Guo, F. , Sun, W.‐H. , Liu, S.‐S. , Tan, H. , & Chen, F. (2019). Antioxidant defense system is responsible for the toxicological interactions of mixtures: A case study on PFOS and PFOA in *Daphnia magna* . Science of the Total Environment, 667, 435–443. 30833242 10.1016/j.scitotenv.2019.02.418

