# Supplementary material for: An Environmentally Relevant Mixture of Perfluorooctanesulfonic Acid and Perfluorohexanesulfonic Acid Does Not Conform to Additivity in Northern Leopard Frogs Exposed Through Metamorphosis
Source: Environ Toxicol Chem. 2022 Nov 2;41(12):3007–16. doi: 10.1002/etc.5486 (PMC9828449; doi:10.1002/etc.5486)

**TABLE & FIGURE CAPTIONS: SUPPLEMENTAL DATA**

**Table S1.** Limits of quantification (LOQs), limits of detection (LODs) and LC-MS/MS conditions used for measuring PFOS and PFHxS in water and whole tadpoles.

| **Coumpond** | **Perfluorohexane sulfonate*** | **Perfluorooctane sulfonate*** |
| --- | --- | --- |
| Acronym | PHHxS | PFOS |
| Formula(as anion) | C6F13O3S- | C8F17O3S- |
| LOQ (µg/L) | 0.06 | 0.05 |
| LOD (µg/L) | 0.02 | 0.02 |
| Internal Standard | 13C3-PFHxS | 13C8-PFOS |
| Internal Standard transition | 402 > 80 | 507 > 80 |
| Native transition (quantifiction) | 399 > 80 | 499 > 80 |
| Native transition (confirmation) | 399 > 99 | 499 > 99 |
| MSMS conditions: Nebulizing Gas Flow:1.5 L/min; DL temperature 250 ⁰C; | | |
| Heat Block Temperature350 ⁰C; Drying Gas Flow 6 L/min; Interface voltage: -3.5kV; DUIS Corona Needle Voltage -3.5 kV; | | |
| Dwell time 30 msec. |  |  |
| * = Both linear and branched isomers available for the compound | |  |
| LOQ: Lowest Limit of Quantification | |  |
| LOD: Lowest Limit of Detection |  |  |

**Table S2.** Raw data summary statistics (mean ± SEM, minimum, maximum) for all variables tested across all treatments at (A) day 31 of exposure, (B) at metamorphic climax (GS 42), (C) across metamorphosis (GS 42- GS 46), and (D) on non-metamorphed tadpoles present at termination of exposures at day 115. ND = Not Detected.

|  |  |  |  |  | |  | |  | |  | | |  | | |  | |  | |  | |  | |  | |  | | |  | | |  | |  | |  | |  | |  | |  | |  | | |  | | |  | |  | |  | |  | |  | | |  |
| --- | --- | --- | --- | --- | --- | --- | --- | --- | --- | --- | --- | --- | --- | --- | --- | --- | --- | --- | --- | --- | --- | --- | --- | --- | --- | --- | --- | --- | --- | --- | --- | --- | --- | --- | --- | --- | --- | --- | --- | --- | --- | --- | --- | --- | --- | --- | --- | --- | --- | --- | --- | --- | --- | --- | --- | --- | --- | --- | --- | --- | --- |
| **A** |  |  |  |  | |  | |  | |  | | | **B** | | |  | |  | |  | |  | |  | |  | | | **C** | | |  | |  | |  | |  | |  | |  | |  | | | **D** | | |  | |  | |  | |  | |  | | |  |
|  | **Day 31** | | | | | | | | | | |  | | | **At Metamorphic Climax (GS 42)** | | | | | | | | | | | | |  | | | **Across Metamorphosis (GS 42 - GS 46)** | | | | | | | | | | | | | | |  | | | **Non-Metamorphed Tadpoles at Day 115** | | | | | | | | | | |  |  |
|  |  | **Unit** | **Treatment** | | **Chemical** | | **Mean ± SEM** | | **Min. Value** | | **Max. Value** | | |  | | |  | | **Unit** | | **Treatment** | | **Mean ± SEM** | | **Min. Value** | | **Max. Value** | | |  | | |  | | **Unit** | | **Treatment** | | **Chemical** | | **Mean ± SEM** | | **Min. Value** | | **Max. Value** | | |  | | |  | | **Treatment** | | **Mean ± SEM** | | **Min. Value** | | **Max. Value** | | |
|  | **Survival** |  | Control | |  | | 0.96 ± 0.01 | | 0.95 | | 1.00 | | |  | | | **Survival to metamorphosis** | |  | | Control | | 0.54 ± 0.05 | | 0.42 | | 0.67 | | |  | | | **Survival through metamorphosis** | |  | | Control | |  | | 0.55 ± 0.12 | | 0.20 | | 0.75 | | |  | | | **Proportion < GS 42** | | Control | | 0.44 ± 0.08 | | 0.27 | | 0.64 | | |
|  |  |  | PFOS 0.5 ppb | |  | | 0.93 ± 0.03 | | 0.85 | | 0.95 | | |  | | |  |  |  |  | PFOS 0.5 ppb | | 0.56 ± 0.02 | | 0.50 | | 0.58 | | |  | | |  |  |  |  | PFOS 0.5 ppb | |  | | 0.63 ± 0.04 | | 0.57 | | 0.71 | | |  | | |  |  | PFOS 0.5 ppb | | 0.39 ± 0.02 | | 0.36 | | 0.45 | | |
|  |  |  | PFOS 1 ppb | |  | | 0.94 ± 0.03 | | 0.85 | | 1.00 | | |  | | |  |  |  |  | PFOS 1 ppb | | 0.63 ± 0.07 | | 0.50 | | 0.83 | | |  | | |  |  |  |  | PFOS 1 ppb | |  | | 0.49 ± 0.08 | | 0.30 | | 0.67 | | |  | | |  |  | PFOS 1 ppb | | 0.38 ± 0.07 | | 0.18 | | 0.50 | | |
|  |  |  | PFHxS 0.5 ppb | |  | | 0.96 ± 0.01 | | 0.95 | | 1.00 | | |  | | |  |  |  |  | PFHxS 0.5 ppb | | 0.69 ± 0.07 | | 0.50 | | 0.83 | | |  | | |  |  |  |  | PFHxS 0.5 ppb | |  | | 0.48 ± 0.11 | | 0.20 | | 0.67 | | |  | | |  |  | PFHxS 0.5 ppb | | 0.35 ± 0.07 | | 0.20 | | 0.55 | | |
|  |  |  | PFHxS 1 ppb | |  | | 0.95 ± 0.02 | | 0.90 | | 1.00 | | |  | | |  |  |  |  | PFHxS 1 ppb | | 0.65 ± 0.04 | | 0.58 | | 0.75 | | |  | | |  |  |  |  | PFHxS 1 ppb | |  | | 0.59 ± 0.09 | | 0.33 | | 0.75 | | |  | | |  |  | PFHxS 1 ppb | | 0.37 ± 0.04 | | 0.25 | | 0.45 | | |
|  |  |  | Mixture | |  | | 0.99 ± 0.01 | | 0.95 | | 1.00 | | |  | | |  |  |  |  | Mixture | | 0.54 ± 0.02 | | 0.50 | | 0.58 | | |  | | |  |  |  |  | Mixture | |  | | 0.47 ± 0.11 | | 0.14 | | 0.67 | | |  | | |  |  | Mixture | | 0.44 ± 0.03 | | 0.36 | | 0.50 | | |
|  | **Gosner Stage** |  | Control | |  | | 30.05 ± 0.35 | | 29.20 | | 30.80 | | |  | | | **Time to metamorphosis** | | days | | Control | | 86.11 ± 3.48 | | 77.00 | | 93.17 | | |  | | | **Length of metamorphosis** | | days | | Control | |  | | 7.92 ± 0.13 | | 7.67 | | 8.25 | | |  | | | **Gosner Stage** | | Control | | 33.02 ± 1.15 | | 30.67 | | 35.86 | | |
|  |  |  | PFOS 0.5 ppb | |  | | 30.00 ± 0.32 | | 29.40 | | 30.80 | | |  | | |  |  |  |  | PFOS 0.5 ppb | | 80.30 ± 1.42 | | 76.29 | | 82.86 | | |  | | |  |  |  |  | PFOS 0.5 ppb | |  | | 8.28 ± 0.30 | | 7.60 | | 9.00 | | |  | | |  |  | PFOS 0.5 ppb | | 35.02 ± 0.21 | | 34.67 | | 35.60 | | |
|  |  |  | PFOS 1 ppb | |  | | 30.45 ± 0.62 | | 29.20 | | 31.20 | | |  | | |  |  |  |  | PFOS 1 ppb | | 84.07 ± 2.36 | | 80.17 | | 90.83 | | |  | | |  |  |  |  | PFOS 1 ppb | |  | | 8.06 ± 0.29 | | 7.50 | | 8.75 | | |  | | |  |  | PFOS 1 ppb | | 34.61 ± 0.46 | | 33.40 | | 35.60 | | |
|  |  |  | PFHxS 0.5 ppb | |  | | 31.25 ± 0.72 | | 30.40 | | 33.40 | | |  | | |  |  |  |  | PFHxS 0.5 ppb | | 83.37 ± 1.42 | | 80.33 | | 87.10 | | |  | | |  |  |  |  | PFHxS 0.5 ppb | |  | | 7.31 ± 0.12 | | 7.00 | | 7.50 | | |  | | |  |  | PFHxS 0.5 ppb | | 31.96 ± 1.92 | | 28.00 | | 35.50 | | |
|  |  |  | PFHxS 1 ppb | |  | | 31.25 ± 0.62 | | 29.80 | | 32.80 | | |  | | |  |  |  |  | PFHxS 1 ppb | | 84.73 ± 2.88 | | 79.57 | | 92.56 | | |  | | |  |  |  |  | PFHxS 1 ppb | |  | | 9.03 ± 0.39 | | 8.17 | | 10.00 | | |  | | |  |  | PFHxS 1 ppb | | 34.47 ± 0.50 | | 33.33 | | 35.75 | | |
|  |  |  | Mixture | |  | | 31.20 ± 0.41 | | 30.40 | | 32.00 | | |  | | |  |  |  |  | Mixture | | 89.73 ± 2.76 | | 85.71 | | 97.71 | | |  | | |  |  |  |  | Mixture | |  | | 7.98 ± 0.24 | | 7.50 | | 8.50 | | |  | | |  |  | Mixture | | 33.88 ± 0.26 | | 33.20 | | 34.40 | | |
|  | **SVL** | mm | Control | |  | | 18.95 ± 0.58 | | 18.00 | | 20.53 | | |  | | | **SVL at metamorphosis** | | mm | | Control | | 25.25 ± 0.23 | | 24.74 | | 25.81 | | |  | | | **Change in SVL** | | mm | | Control | |  | | 1.25 ± 0.38 | | 0.43 | | 2.21 | | |  | | |  | |  | |  | |  | |  | | |
|  |  |  | PFOS 0.5 ppb | |  | | 18.66 ± 0.60 | | 17.70 | | 20.36 | | |  | | |  |  |  |  | PFOS 0.5 ppb | | 24.95 ± 0.21 | | 24.38 | | 25.40 | | |  | | |  |  |  |  | PFOS 0.5 ppb | |  | | 1.53 ± 0.29 | | 1.05 | | 2.36 | | |  | | |  | |  | |  | |  | |  | | |
|  |  |  | PFOS 1 ppb | |  | | 19.69 ± 0.80 | | 17.35 | | 20.74 | | |  | | |  |  |  |  | PFOS 1 ppb | | 24.59 ± 0.35 | | 24.13 | | 25.61 | | |  | | |  |  |  |  | PFOS 1 ppb | |  | | 1.23 ± 0.13 | | 0.89 | | 1.49 | | |  | | |  | |  | |  | |  | |  | | |
|  |  |  | PFHxS 0.5 ppb | |  | | 20.97 ± 0.64 | | 20.27 | | 22.89 | | |  | | |  |  |  |  | PFHxS 0.5 ppb | | 24.22 ± 0.17 | | 23.72 | | 24.45 | | |  | | |  |  |  |  | PFHxS 0.5 ppb | |  | | 1.43 ± 0.30 | | 0.56 | | 1.96 | | |  | | |  | |  | |  | |  | |  | | |
|  |  |  | PFHxS 1 ppb | |  | | 20.21 ± 0.62 | | 19.06 | | 21.89 | | |  | | |  |  |  |  | PFHxS 1 ppb | | 24.89 ± 0.16 | | 24.53 | | 25.16 | | |  | | |  |  |  |  | PFHxS 1 ppb | |  | | 0.50 ± 0.25 | | 0.00 | | 1.17 | | |  | | |  | |  | |  | |  | |  | | |
|  |  |  | Mixture | |  | | 20.00 ± 0.64 | | 18.79 | | 21.43 | | |  | | |  |  |  |  | Mixture | | 24.37 ± 0.44 | | 23.47 | | 25.50 | | |  | | |  |  |  |  | Mixture | |  | | 1.17 ± 0.27 | | 0.67 | | 1.68 | | |  | | |  | |  | |  | |  | |  | | |
|  | **Mass** | g | Control | |  | | 1.17 ± 0.12 | | 0.86 | | 1.42 | | |  | | | **Mass at metamorphosis** | | g | | Control | | 2.41 ± 0.13 | | 2.14 | | 2.75 | | |  | | | **Change in Mass** | | g | | Control | |  | | -1.00 ± 0.04 | | -1.07 | | -0.88 | | |  | | |  | |  | |  | |  | |  | | |
|  |  |  | PFOS 0.5 ppb | |  | | 0.98 ± 0.08 | | 0.85 | | 1.17 | | |  | | |  |  |  |  | PFOS 0.5 ppb | | 2.26 ± 0.05 | | 2.14 | | 2.39 | | |  | | |  |  |  |  | PFOS 0.5 ppb | |  | | -0.94 ± 0.05 | | -1.04 | | -0.83 | | |  | | |  | |  | |  | |  | |  | | |
|  |  |  | PFOS 1 ppb | |  | | 1.18 ± 0.12 | | 0.89 | | 1.44 | | |  | | |  |  |  |  | PFOS 1 ppb | | 2.22 ± 0.12 | | 2.07 | | 2.57 | | |  | | |  |  |  |  | PFOS 1 ppb | |  | | -0.88 ± 0.02 | | -0.95 | | -0.85 | | |  | | |  | |  | |  | |  | |  | | |
|  |  |  | PFHxS 0.5 ppb | |  | | 1.29 ± 0.07 | | 1.15 | | 1.46 | | |  | | |  |  |  |  | PFHxS 0.5 ppb | | 2.15 ± 0.06 | | 2.00 | | 2.26 | | |  | | |  |  |  |  | PFHxS 0.5 ppb | |  | | -0.80 ± 0.05 | | -0.93 | | -0.67 | | |  | | |  | |  | |  | |  | |  | | |
|  |  |  | PFHxS 1 ppb | |  | | 1.22 ± 0.09 | | 1.02 | | 1.48 | | |  | | |  |  |  |  | PFHxS 1 ppb | | 2.27 ± 0.05 | | 2.15 | | 2.40 | | |  | | |  |  |  |  | PFHxS 1 ppb | |  | | -0.88 ± 0.03 | | -0.94 | | -0.82 | | |  | | |  | |  | |  | |  | |  | | |
|  |  |  | Mixture | |  | | 1.21 ± 0.11 | | 0.97 | | 1.47 | | |  | | |  |  |  |  | Mixture | | 2.12 ± 0.14 | | 1.85 | | 2.48 | | |  | | |  |  |  |  | Mixture | |  | | -0.97 ± 0.08 | | -1.16 | | -0.79 | | |  | | |  | |  | |  | |  | |  | | |
|  | **SMI** |  | Control | |  | | 1.10 ± 0.05 | | 1.00 | | 1.23 | | |  | | | **SMI at metamorphosis** | |  | | Control | | 2.34 ± 0.05 | | 2.23 | | 2.45 | | |  | | | **Change in SMI** | |  | | Control | |  | | -0.95 ± 0.07 | | -1.13 | | -0.85 | | |  | | |  | |  | |  | |  | |  | | |
|  |  |  | PFOS 0.5 ppb | |  | | 0.99 ± 0.02 | | 0.94 | | 1.03 | | |  | | |  |  |  |  | PFOS 0.5 ppb | | 2.33 ± 0.03 | | 2.29 | | 2.40 | | |  | | |  |  |  |  | PFOS 0.5 ppb | |  | | -1.00 ± 0.09 | | -1.21 | | -0.83 | | |  | | |  | |  | |  | |  | |  | | |
|  |  |  | PFOS 1 ppb | |  | | 0.98 ± 0.04 | | 0.88 | | 1.08 | | |  | | |  |  |  |  | PFOS 1 ppb | | 2.37 ± 0.03 | | 2.30 | | 2.42 | | |  | | |  |  |  |  | PFOS 1 ppb | |  | | -0.99 ± 0.08 | | -1.19 | | -0.86 | | |  | | |  | |  | |  | |  | |  | | |
|  |  |  | PFHxS 0.5 ppb | |  | | 0.91 ± 0.04 | | 0.79 | | 1.00 | | |  | | |  |  |  |  | PFHxS 0.5 ppb | | 2.43 ± 0.06 | | 2.32 | | 2.60 | | |  | | |  |  |  |  | PFHxS 0.5 ppb | |  | | -1.09 ± 0.10 | | -1.34 | | -0.88 | | |  | | |  | |  | |  | |  | |  | | |
|  |  |  | PFHxS 1 ppb | |  | | 0.98 ± 0.02 | | 0.95 | | 1.02 | | |  | | |  |  |  |  | PFHxS 1 ppb | | 2.31 ± 0.03 | | 2.23 | | 2.36 | | |  | | |  |  |  |  | PFHxS 1 ppb | |  | | -0.93 ± 0.14 | | -1.21 | | -0.56 | | |  | | |  | |  | |  | |  | |  | | |
|  |  |  | Mixture | |  | | 0.99 ± 0.02 | | 0.95 | | 1.06 | | |  | | |  |  |  |  | Mixture | | 2.33 ± 0.01 | | 2.31 | | 2.36 | | |  | | |  |  |  |  | Mixture | |  | | -0.94 ± 0.06 | | -1.09 | | -0.79 | | |  | | |  | |  | |  | |  | |  | | |
|  | **Body Burden** | ng g^-1^ dw | Control | | PFOS | | 18.45 ± 2.31 | | 12.46 | | 23.74 | | |  | | |  | |  | |  | |  | |  | |  | | |  | | | **Body Burden at GS 46** | | ng g^-1^ dw | | Control | | PFOS | | 1.76 ± 1.08 | | ND | | 4.89 | | |  | | |  | |  | |  | |  | |  | | |
|  |  |  |  | | PFHxS | | 1.08 ± 0.44 | | 0.27 | | 2.31 | | |  | | |  | |  | |  | |  | |  | |  | | |  | | |  |  |  |  |  | | PFHxS | | ND | | -- | | -- | | |  | | |  | |  | |  | |  | |  | | |
|  |  |  | PFOS 0.5 ppb | | PFOS | | 324.86 ± 37.76 | | 215.24 | | 386.78 | | |  | | |  | |  | |  | |  | |  | |  | | |  | | |  |  |  |  | PFOS 0.5 ppb | | PFOS | | 363.33 ± 18.19 | | 311.18 | | 394.46 | | |  | | |  | |  | |  | |  | |  | | |
|  |  |  |  | | PFHxS | | 0.44 ± 0.17 | | 0.16 | | 0.92 | | |  | | |  | |  | |  | |  | |  | |  | | |  | | |  |  |  |  |  | | PFHxS | | ND | | -- | | -- | | |  | | |  | |  | |  | |  | |  | | |
|  |  |  | PFOS 1 ppb | | PFOS | | 755.40 ± 58.51 | | 588.72 | | 849.20 | | |  | | |  | |  | |  | |  | |  | |  | | |  | | |  |  |  |  | PFOS 1 ppb | | PFOS | | 829.54 ± 134.17 | | 589.56 | | 1142.77 | | |  | | |  | |  | |  | |  | |  | | |
|  |  |  |  | | PFHxS | | 0.65 ± 0.52 | | ND | | 1.70 | | |  | | |  | |  | |  | |  | |  | |  | | |  | | |  |  |  |  |  | | PFHxS | | 3.63 | | ND | | 3.63 | | |  | | |  | |  | |  | |  | |  | | |
|  |  |  | PFHxS 0.5 ppb | | PFOS | | 7.41 ± 1.51 | | 3.18 | | 10.12 | | |  | | |  | |  | |  | |  | |  | |  | | |  | | |  |  |  |  | PFHxS 0.5 ppb | | PFOS | | 2.04 | | ND | | 2.04 | | |  | | |  | |  | |  | |  | |  | | |
|  |  |  |  | | PFHxS | | 4.04 ± 0.20 | | 3.60 | | 4.52 | | |  | | |  | |  | |  | |  | |  | |  | | |  | | |  |  |  |  |  | | PFHxS | | 3.42 ± 1.72 | | ND | | 5.42 | | |  | | |  | |  | |  | |  | |  | | |
|  |  |  | PFHxS 1 ppb | | PFOS | | 12.61 ± 0.66 | | 10.79 | | 13.89 | | |  | | |  | |  | |  | |  | |  | |  | | |  | | |  |  |  |  | PFHxS 1 ppb | | PFOS | | 0.41 | | ND | | 0.41 | | |  | | |  | |  | |  | |  | |  | | |
|  |  |  |  | | PFHxS | | 4.88 ± 0.22 | | 4.31 | | 5.28 | | |  | | |  | |  | |  | |  | |  | |  | | |  | | |  |  |  |  |  | | PFHxS | | 11.03 ± 4.30 | | 3.98 | | 23.54 | | |  | | |  | |  | |  | |  | |  | | |
|  |  |  | Mixture | | PFOS | | 650.80 ± 199.07 | | 306.78 | | 1167.05 | | |  | | |  | |  | |  | |  | |  | |  | | |  | | |  |  |  |  | Mixture | | PFOS | | 413.60 ± 53.76 | | 254.95 | | 491.18 | | |  | | |  | |  | |  | |  | |  | | |
|  |  |  |  | | PFHxS | | 1.83 ± 0.31 | | 1.40 | | 2.77 | | |  | | |  | |  | |  | |  | |  | |  | | |  | | |  |  |  |  |  | | PFHxS | | 2.90 ± 1.70 | | ND | | 6.57 | | |  | | |  | |  | |  | |  | |  | | |
|  |  |  |  | |  | |  | |  | |  | | |  | | |  | |  | |  | |  | |  | |  | | |  | | | **BCF** | |  | | PFOS 0.5 ppb | |  | | 687.58 ± 71.32 | | 475.83 | | 774.09 | | |  | | |  | |  | |  | |  | |  | | |
|  |  |  |  | |  | |  | |  | |  | | |  | | |  | |  | |  | |  | |  | |  | | |  | | |  |  |  |  | PFOS 1 ppb | |  | | 886.09 ± 137.49 | | 617.99 | | 1231.44 | | |  | | |  | |  | |  | |  | |  | | |
|  |  |  |  | |  | |  | |  | |  | | |  | | |  | |  | |  | |  | |  | |  | | |  | | |  |  |  |  | PFHxS 0.5 ppb | |  | | 7.92 ± 3.96 | | 0.00 | | 11.92 | | |  | | |  | |  | |  | |  | |  | | |
|  |  |  |  | |  | |  | |  | |  | | |  | | |  | |  | |  | |  | |  | |  | | |  | | |  |  |  |  | PFHxS 1 ppb | |  | | 9.39 ± 3.71 | | 3.30 | | 20.19 | | |  | | |  | |  | |  | |  | |  | | |
|  |  |  |  | |  | |  | |  | |  | | |  | | |  | |  | |  | |  | |  | |  | | |  | | |  |  |  |  | Mixture | | PFOS | | 947.31 ± 81.93 | | 760.03 | | 1127.20 | | |  | | |  | |  | |  | |  | |  | | |
|  |  |  |  | |  | |  | |  | |  | | |  | | |  | |  | |  | |  | |  | |  | | |  | | |  |  |  |  |  | | PFHxS | | 5.29 ± 3.09 | | 0.00 | | 11.64 | | |  | | |  | |  | |  | |  | |  | | |
|  |  |  |  | |  | |  | |  | |  | | |  | | |  | |  | |  | |  | |  | |  | | |  | | | **Total Survival Throughout Exposure Period** | |  | | Control | |  | | 0.79 ± 0.04 | | 0.73 | | 0.90 | | |  | | |  | |  | |  | |  | |  | | |
|  |  |  |  | |  | |  | |  | |  | | |  | | |  | |  | |  | |  | |  | |  | | |  | | |  |  |  |  | PFOS 0.5 ppb | |  | | 0.76 ± 0.03 | | 0.69 | | 0.82 | | |  | | |  | |  | |  | |  | |  | | |
|  |  |  |  | |  | |  | |  | |  | | |  | | |  | |  | |  | |  | |  | |  | | |  | | |  |  |  |  | PFOS 1 ppb | |  | | 0.69 ± 0.08 | | 0.45 | | 0.82 | | |  | | |  | |  | |  | |  | |  | | |
|  |  |  |  | |  | |  | |  | |  | | |  | | |  | |  | |  | |  | |  | |  | | |  | | |  |  |  |  | PFHxS 0.5 ppb | |  | | 0.69 ± 0.11 | | 0.40 | | 0.91 | | |  | | |  | |  | |  | |  | |  | | |
|  |  |  |  | |  | |  | |  | |  | | |  | | |  | |  | |  | |  | |  | |  | | |  | | |  |  |  |  | PFHxS 1 ppb | |  | | 0.78 ± 0.11 | | 0.50 | | 1.00 | | |  | | |  | |  | |  | |  | |  | | |
|  |  |  |  | |  | |  | |  | |  | | |  | | |  | |  | |  | |  | |  | |  | | |  | | |  |  |  |  | Mixture | |  | | 0.73 ± 0.10 | | 0.50 | | 0.90 | | |  | | |  | |  | |  | |  | |  | | |

**Figure S1.** Change in SMI (mean ± SEM) across metamorphosis (GS 42 – GS 46) in Northern leopard frog tadpoles. Shaded triangle within the mixture treatment represents the expected SMI based on additive effects of PFOS and PFHxS at 0.5 ppb at each timepoint.
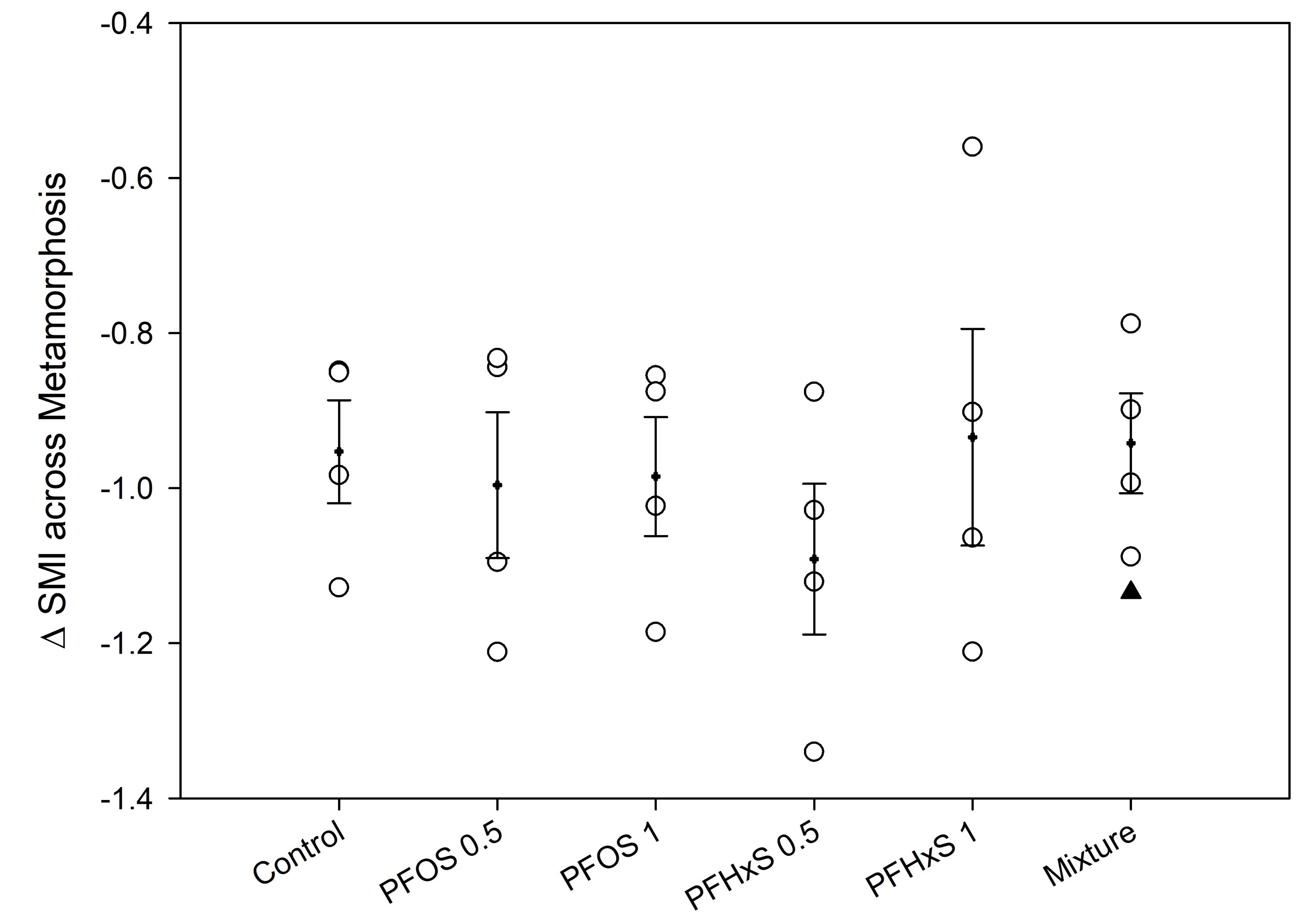

Supplement: Supplementary file 1 — Supporting file. [file ETC-41-3007-s002.docx]
